# Normal modes analysis and surface electrostatics of haemagglutinin proteins as fingerprints for high pathogenic type A influenza viruses

**DOI:** 10.1186/s12859-020-03563-w

**Published:** 2020-08-21

**Authors:** Irene Righetto, Francesco Filippini

**Affiliations:** Department of Biology, University of Padua, Synthetic Biology and Biotechnology Unit, via U. Bassi 58/B, 35131 Padova, Italy

**Keywords:** Haemagglutinin, Avian influenza virus, H5N1, HPAI, LPAI, Homology modeling, Electrostatic distance, Normal modes analysis

## Abstract

**Background:**

Type A influenza viruses circulate and spread among wild birds and mostly consist of low pathogenic strains. However, fast genome variation timely results in the insurgence of high pathogenic strains, which when infecting poultry birds may cause a million deaths and strong commercial damage. More importantly, the host shift may concern these viruses and sustained human-to-human transmission may result in a dangerous pandemic outbreak. Therefore, fingerprints specific to either low or high pathogenic strains may represent a very important tool for global surveillance.

**Results:**

We combined Normal Modes Analysis and surface electrostatic analysis of a mixed strain dataset of influenza A virus haemagglutinins from high and low pathogenic strains in order to infer specific fingerprints. Normal Modes Analysis sorted the strains in two different, homogeneous clusters; sorting was independent of clades and specific instead to high vs low pathogenicity. A deeper analysis of fluctuations and flexibility regions unveiled a special role for the 110-helix region. Specific sorting was confirmed by surface electrostatics analysis, which further allowed to focus on regions and mechanisms possibly crucial to the low-to-high transition.

**Conclusions:**

Evidence from previous work demonstrated that changes in surface electrostatics are associated with the evolution and spreading of avian influenza A virus clades, and seemingly involved also in the avian to mammalian host shift. This work shows that a combination of electrostatics and Normal Modes Analysis can also identify fingerprints specific to high and low pathogenicity. The possibility to predict which specific mutations may result in a shift to high pathogenicity may help in surveillance and vaccine development.

## Background

RNA viruses are characterized by high mutation rate and thus by high genetic variability; as a consequence, viral populations consist of a mixture of genetically related variants, rather than of a defined genome sequence. Influenza A viruses cause respiratory infections, ranging from asymptomatic or moderate disease in a healthy population, to deadly in weak individuals. Different subtypes of such viruses may be specific to either birds or mammals, and they infect billions of individuals per year. In humans, seasonal epidemic outbreaks by ‘standard’ viral strains may cause up to 500.000 yearly deaths worldwide ([1], and data from WHO and CDC [2, 3]). However, death occurrence may dramatically increase when pandemic outbreaks concern highly pathogenic strains. In the past century, severe influenza A outbreaks occurred in 3 years (1918, 1957 and 1968), resulting in a huge death toll (~ 100 million people worldwide [4]), even higher than the aggregate effect of the two world wars. Wild ducks are the largest reservoir of avian influenza (AI) viruses, which sporadically also infect domestic birds. Some subtypes of AI viruses - especially H5 and H7 [5] - have the potential to evolve into high pathogenic (HPAI) forms from low pathogenic (LPAI) precursors. In wild birds, HPAI viruses are not associated with high mortality or severe disease; instead, occurrence of LPAI to HPAI shift in poultry may cause massive death and economic damage [6–9]. Sometimes, AI viruses may perform ‘host jump’ [10–12] and infect domestic mammalian species (e.g. swine, cats, dogs, horses and also humans). Human cases naturally infected by AI viruses especially spanned subtypes H5, H7 and H9 and (to a minor extent) H6 and H10 [13–17]. Therefore, sudden LPAI to HPAI shift and possible host jump represent a recurring, dangerous threat to both the human health and poultry industry. This is difficult to address by seasonal vaccines, because of the ‘escape’ strategy of viruses (antigenic drift), favoured in turn by the extremely high frequency of variation of their antigens. In such a context, research organizations and teams worldwide have tried to shed more light on relevant molecular mechanisms and to infer as more information as possible on fingerprints that might help to predict trends in viral clade evolution and spreading, antigenic drift, pathogenicity shift and host jump.

A central role in all these phenomena is played by the spike protein haemagglutinin (HA), which is thus the most investigated AI virus gene/protein. HA is the main viral surface antigen hence the major player in stimulating the antibody response and in antigenic drift [18]. HA is crucial to the attachment and penetration into the host cell and thus it acts as a key contributor to change host specificity in AI viral infection [19]. Structurally, HA mature monomers consist of chains HA1 and HA2, which are produced by proteolytic cleavage of the unfolded precursor. Such cleavage is crucial to HA1 trimerisation [18] and it is also involved in LPAI to HPAI shift, as the emergence of HPAI viruses has been most often associated to the insertion or substitution of basic amino acids at the cleavage site [20, 21].

Indeed, the overall classification of AI viruses is based on serological and phylogenetic differences in HA and neuraminidase [22]; e.g. H9N2 viruses have H9 subtype HA combined to N2 type neuraminidase. Both sensitivity to vaccines and antigenic drift depend on changes in HA serological specificity, which in turn depends on the variation of epitopes recognized by each specific antibody, rather than on the extent of sequence divergence. Therefore, we recently performed a structural bioinformatics analysis of the surface variation among different AI viral subtypes, as well among different clades from the same subtype. In particular, the availability of a number of structural templates from different HA subtypes allowed us to perform comparative analyses. This led to identify subtype- and subregion-specific variation in surface electrostatics, especially concerning the HA ‘head’ named receptor binding domain (RBD) [23]. Moreover, a deeper analysis of variation among H5N1 clades and subclades unveiled electrostatic fingerprints, which relate to both the evolution and spreading of clades, suggesting that surface charge redistribution is likely involved in antigenic drift events [23]. The hypothesis that such fingerprinting system could be limited to the H5 subtype was then excluded by an extensive and detailed phylogenetic and structural comparison of H9 viruses, demonstrating that electrostatic variation of HA is a hallmark of the AI viruses evolution [24]. In particular, phylogenetic analyses of H9 viruses isolated from wild birds and poultry reliably identified five main groups and comparison of their electrostatic features showed congruence between phylogenetic clustering and surface fingerprints, which in turn relate to well-known HA sites involved in the modulation of immune escape and host specificity [24]. Indeed, spike proteins such as HA do not interact only with antibodies and thus, in addition to determining antigenic drift, surface feature variation can also influence interaction with cell receptors hence host specificity. This prompted further analyses on the variation of HA surface features among AI viruses isolated from different avian and mammalian (including human) hosts, aimed to investigate clustering and eventual fingerprints among representative pandemic (H5 and H7) and non-pandemic (H4 and H6) AI viral subtypes. This analysis unveiled preferential sorting (even if it was not ‘100% specific’) of the viruses isolated from mammalian/human hosts among the electrostatic clusters of a subtype [25]. This suggested that electrostatic fingerprints are involved also in host jumping and thus they can help shedding more light on it, but they cannot explain alone the whole mechanism.

Even though the emergence of high pathogenicity viruses has been associated with changes at the HA cleavage site [20, 21, 26], other mechanisms are likely involved. Indeed, in H5 and H7 viruses, the LPAI to HPAI shift was found to be associated with variation in glycosylation sites at the haemagglutinin RBD [27]. More recently, in H5N1 isolates from Hong Kong, HPAI and LPAI viruses were found to be expressed and cleaved in similar amounts, while changes at the 110-helix region of the vestigial esterase subdomain (VED) of the RBD resulted in modulating the pH of HA activation and thus pathogenicity [28]. HA activation and interaction with elements of the respiratory system are of course influenced by HA protein dynamics and by surface features, and this in turn is likely to modulate virus pathogenicity. This prompted us to check whether variation of the electrostatic features might help finding fingerprints able to distinguish HPAI from LPAI viruses, and to provide more evidence on the molecular mechanisms involved in this transition. In addition, we performed Normal Modes Analysis (NMA) of the same HPAI and LPAI virus strains datasets used for the electrostatic analysis, and report here that we could validate every specific fingerprint by these such two different and independent algorithmic approaches.

## Methods

### Structural templates and target sequences

Two structures from the Protein Data Bank (PDB) were used as templates for modeling either the selected HPAI or LPAI target sequences: PDB 3S11, from viral strain A/Goose/Guangdong/1/1996 (HPAI H5N1) and PDB 5YKC from viral strain A/chicken/Taiwan/0502/2012 (LPAI H5N1). UniProtKb accession codes (AC) for HPAI and LPAI H5N1 target sequences modeled by homology modeling, corresponding viral strains (VS) and a list of supporting references are reported in Supplementary Table S1.

### Structural superpositions, homology modeling, model refinement, and quality check

According to previous methodological set up [23], structural superpositions were performed and viewed using UCSF Chimera [29] v. 1.13.1 (free download from [30]). Target protein sequences were modeled by homology on the best available structure templates using SWISS-MODEL ([31], accessed August 2019). Then, model structures were refined using SCWRL [32, 33]. Model quality was checked via the QMEAN server ([34] accessed August 2019).

### Electrostatic surface analysis

Isopotential contours were calculated using UCSF Chimera 1.13.1, which allows for connecting - through Opal web server - to the Adaptive Poisson-Boltzmann Solver (APBS) server ([35] accessed October 2019). The isopotential contours were then plotted at ±1k_B_T/e. PDB2PQR [36, 37] was used to assign partial charges and van der Waals radii according to the PARSE force field [38]. Interior ε_p_ = 2 and ε_s_ = 78.5 were chosen for respectively the protein and the solvent [39–41], T = 298.15 K. Probe radius for dielectric surface and ion accessibility surface were set to be *r* = 1.4 Å and *r* = 2.0 Å, respectively. Rigid-body superposition was performed and the electrostatic potential was computed using Chimera 1.13.1. Electrostatic distance (ED) was calculated using the Hodgkin index and the Carbo index at the WebPIPSA server ([42] accessed October 2019).

### Single and comparative normal modes analysis (NMA)

Single and comparative NMA analyses were performed using the WebNM@ server ([43] accessed October 2019). This tool is able to calculate the low-frequency normal modes by building the coarse-grained Elastic Network Model (ENM) of a submitted pdb file [44, 45]. This way, the protein is represented as a string of beads of C_α_ atoms, interacting following formula F_a_:

$$ {U}_{ij}(r)=\frac{k_{ij}}{2}{\left(\left|{r}_i-{r}_j\right|-\left|{r}_i^0-{r}_j^0\right|\right)}^2 $$

where r_i_ and r_j_ are the positions of residues I and j in the current conformation of the protein, and the superscript ^0^ denotes the equilibrium conformation; k_ij_ is the force constant for the spring connecting residues I and j. In the single mode, this software calculates the dynamic cross-correlation matrices (DCCM) [44], which help to identify the correlated and anticorrelated motions [45]. The coupling between two C_α_ atoms i and j in the DCCM is defined by formula F_b_:

$$ {C}_{ij}=\frac{\sum_{m=1}^M\frac{1}{\gamma_m}\left[{X}_m\right]i\left[{X}_m\right]j}{\sqrt{\sum_{m=1}^M\frac{1}{\gamma_m}\left[{X}_m\right]i\left[{X}_m\right]i}\kern0.5em \sqrt{\sum_{m=1}^M\frac{1}{\gamma_m}\left[{X}_m\right]j\left[{X}_m\right]j}} $$

Here, *X*_*m*_ and *γ*_*m*_ describe the eigenvectors and eigenvalues of the mth normal mode. In this work, default settings for WebNM@ are used. Graphs showing the C_α_ atoms fluctuations are reported in the supplementary material, as they can help understanding the proteins’ dynamics in different modes. PDBeFOLD [46] was used to perform structural alignments, requested by the WebNM@ server (accessed October 2019). This kind of analysis is useful to investigate the dynamic similarity in terms of Bhattacharyya coefficient (BC) [43] and Root Mean Square Inner Product (RMSIP) [43]. The BC measures the dynamical similarity between proteins by comparing their covariance matrices, obtained from the normal modes of the conserved parts of the considered proteins. BC values range from 0 to 1. BC of 1 represents the maximum overlap (or dynamical similarity) between the collective dynamics of the aligned proteins. The RMSIP allows for quantitative comparison of C_α_ atoms fluctuations between proteins. This index was computed for the lowest normal modes using equation F_c_:

$$ RMSIP=\sqrt{\frac{1}{n}\left[\sum \limits_{i=1}^n\sum \limits_{j=1}^n{\left({X}_i{Y}_i\right)}^2\right]} $$

The RMSIP values range from 0 to 1; RMSIP of 1 represents maximum similarity in C_α_ atoms fluctuations between compared proteins. WebNM@ also provides the graph of the C_α_ atoms fluctuations, where the normalized squared C_α_ atoms fluctuations for each protein are calculated as the sum of the displacement of each C_α_ atom along with the lowest modes [43]. The fluctuations are the sum of the C_α_ atoms displacements in each mode, weighted by the inverse of their corresponding eigenvalues. The first 200 modes are used to carry out these calculations. Flexible protein regions can be inferred by inspecting the peaks of the fluctuations graph.

## Results and discussion

### Preliminary comparison of a mixed dataset of AI viruses from HPAI and LPAI strains

A number of H5N1 haemagglutinin sequences were collected from UniProtKB [47] and the Influenza Research Database (IRD) [48, 49] (both accessed August 2019). Ten sequences representative for HPAI strains, and ten for LPAI ones were selected considering next computational constraints (i.e. restriction to 20 sequences max in NMA steps with WebNM@). According to previous work [23], corresponding structural models were obtained via Homology Modeling with high confidence, because of the very high identity values (over 90%) among target sequences and templates. This notwithstanding, structural models were further refined (see methods), resulting in all instances in high quality values (i.e. inside or close to the blue region in QMEAN interface), no atom clashes and in Local Quality Estimate being high as well, in all protein subregions. Details on the 20 protein sequences, viral strains they are derived from, structural modeling and refinement are reported in the methods section and in Supplementary Table S1. Structure pair-wise superpositions were performed using UCSF Chimera [29] to calculate Root Mean Square Deviation (RMSD). PDBeFOLD [46] was used to perform structural alignments. Within each sub-dataset, the HPAI and LPAI sequences and their corresponding structures were compared in terms of % identity and RMSD values, as % identity is commonly used as an index for ‘sequence divergence’ [50], while RMSD of two superposed structures indicates the ‘structural divergence’ from one another [51]. The sequence identities across HPAI and LPAI HA monomers, as well as RMSD values as inferred from PDBeFOLD, are presented in Supplementary Figure S1. The very high sequence identity values satisfied the parameters reported in previous analyses [23–25] and the RMSD values within both HPAI and LPAI groups were observed to be largely < 1 Å, highlighting the strong structural conservation which is commonly observed within each viral subtype. Similar results were found when repeating the comparison among whole trimers or restricting it to the most antigenic parts of HA, i.e. to the RBD region (not shown). Indeed, when comparing haemagglutinins from different HA subtypes, % identity ranges 41–49% [25], while in this comparison all sequences belong to H5N1, explaining % identity values > 95% and subsequent finding of RMSD values close to zero. When comparing HPAI to LPAI sequences or structures from this dataset, % identity values or RMSD do not change meaningfully (not shown), suggesting that minor sequence/structure changes are responsible for the different pathogenicity features, and that analyses deeper than simple, direct or multiple, sequence or structure comparison are needed. This is not surprising, as in proteins (and in their domains) specific molecular interactions or processes crucial to function and pathogenicity may be mediated by very small motifs, and often even a single amino acid change can alter the motif properties. Therefore, in order to infer specific fingerprints, NMA was performed.

### NMA of HA RBDs from HPAI and LPAI strains: Bhattacharyya coefficient (BC) heatmap

The fluctuation profiles for C_α_ atoms of all 20 representative proteins were obtained from the NMA study. The comparative NMA analysis of HA was performed for RBDs, monomers, and trimers in order to find conservation (or differences) of dynamics across HPAI and LPAI strains. Results for trimers and monomers are presented in supplementary Figures S2 to S4, while NMA comparison of RBDs is presented and discussed hereafter. This depends on the fact that relevant differences among the HPAI and LPAI viruses are already reported for other HA subregions, such as e.g. the proteolytic cleavage site, and this, however, could not entirely explain the difference in pathogenicity, as already discussed in the introduction section. Special attention to the RBD depends on evidence that just this subdomain mediates the most of HA interactions, as it contains the major determinants for antigenic variation and antigenic drift [18, 51], as well as others likely involved in host jump [19, 25]. Moreover, slight variation in the RBD surface features is a fingerprint for clades evolution and spreading in both H5 and H9 subtypes [23, 24].

**Fig. 1:**
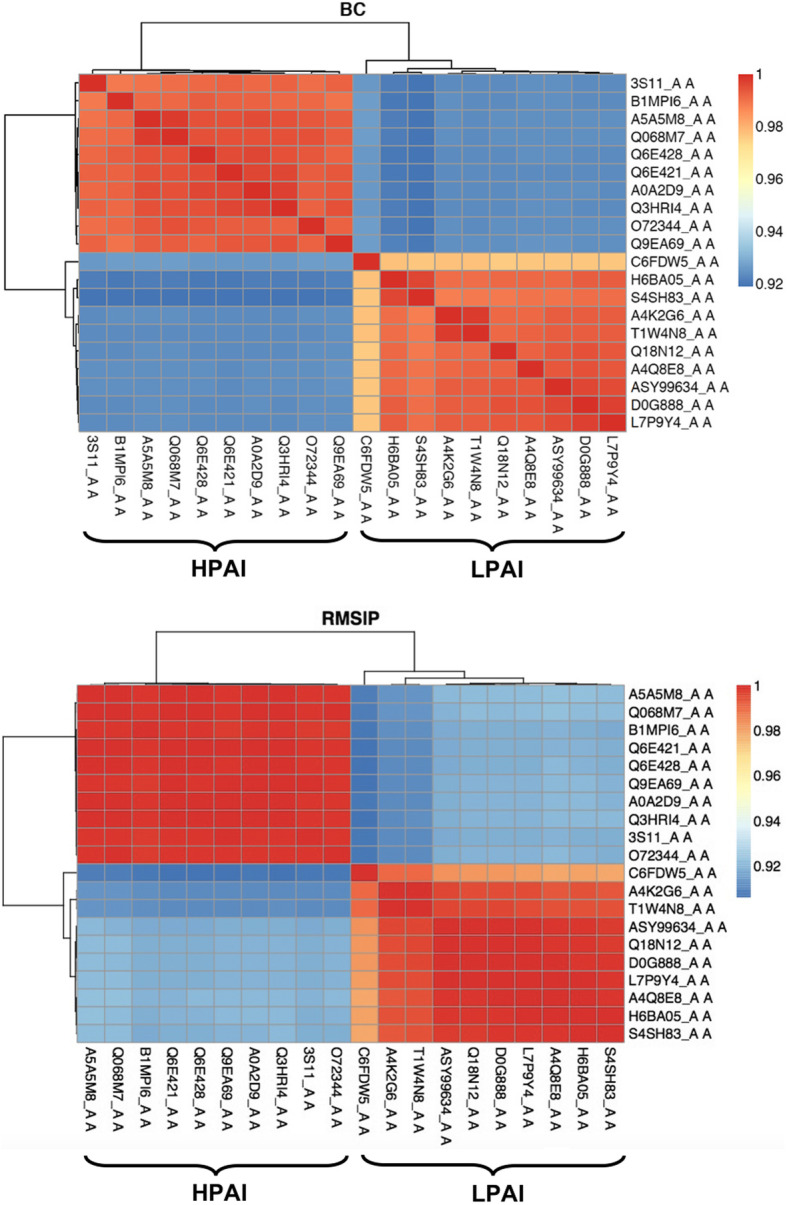
Bhattacharyya coefficient (BC) heatmap (upper panel) and Root Mean Square Inner Product (RMSIP) heatmap (lower panel) for HPAI and LPAI RBDs. The Uniprot AC (and only one PDB AC) of the proteins are reported at both the x- and y-axis, while BC or RMSIP values range in the figures are colour coded from the lowest (blue) to the highest (red)

The Bhattacharyya coefficient (BC) heatmap for HPAI and LPAI RBDs is presented in Fig. 1, where red shading (BC of 1) represents the maximum similarity in dynamics of proteins used for comparison, and blue shading indicates the least similar dynamics. In Fig. 1, all BC values (upper panel) are very high, as the lowest score is 0.92, meaning that the overall fluctuation is quite similar in all strains; a similar picture is presented (lower panel) by the Root Mean Square Inner Product (RMSIP). It has to be stressed that the 20 sequences analysed in this work were compared (each being used as the query sequence for blastp in blast2sequences mode) to those representative for the ten H5N1 clades [23] (used altogether as the multifasta subject database). This showed that 13 such sequences belong to clade 0 (all sequences from LPAI viruses and three from HPAI ones), six HPAI sequences to clade 3 and one HPAI sequence to clade 5. It is noteworthy that NMA separates the 20 representative RBDs into only two clusters related to the RBD dynamics, and that clustering between HPAI and LPAI viruses is specific and sharp. Indeed, if separation would depend on homology, three clusters (each containing sequences from one clade) would have been apparent. Instead, NMA sorted the sequences only based on the type of pathogenicity, as e.g. the three HPAI sequences from clade 0 viruses sorted altogether with HPAI sequences from clades 3 and 5 into the “HPAI cluster” (which is characterized by cold colours for higher divergency, according to the presence of sequences from different clades), separately from the LPAI sequences from clade 0, which sorted altogether into the (more homogeneous and thus with warm colours) “LPAI cluster”. Since the BC range is narrow and all values are high, we can infer that HPAI and LPAI viruses dynamics differ by a very little difference. Taking a look at the heatmap, we can notice that proteins within a group (HPAI or LPAI) are very similar in terms of dynamics. Even though statistical support is intrinsic to the WebNMA tool and thus protein clustering in the heatmap is statistically meaningful, we also checked whether clustering of NMA dynamic behavior could depend on the structural template selected for modeling rather than reflecting different features of the two ensembles of sequences. In order to addres this point, all 20 sequences were modeled on the LPAI template (5YKC). As shown in supplementary Figure S5, homogeneous template modeling did not change the clustering at all. When repeating the analysis with homogeneous HPAI template (3S11), once again LPAI and HPAI modeled structures sorted separately in the same observed way.

### NMA of HA RBDs from HPAI and LPAI strains: dynamic cross-correlation matrices

**Fig. 2:**
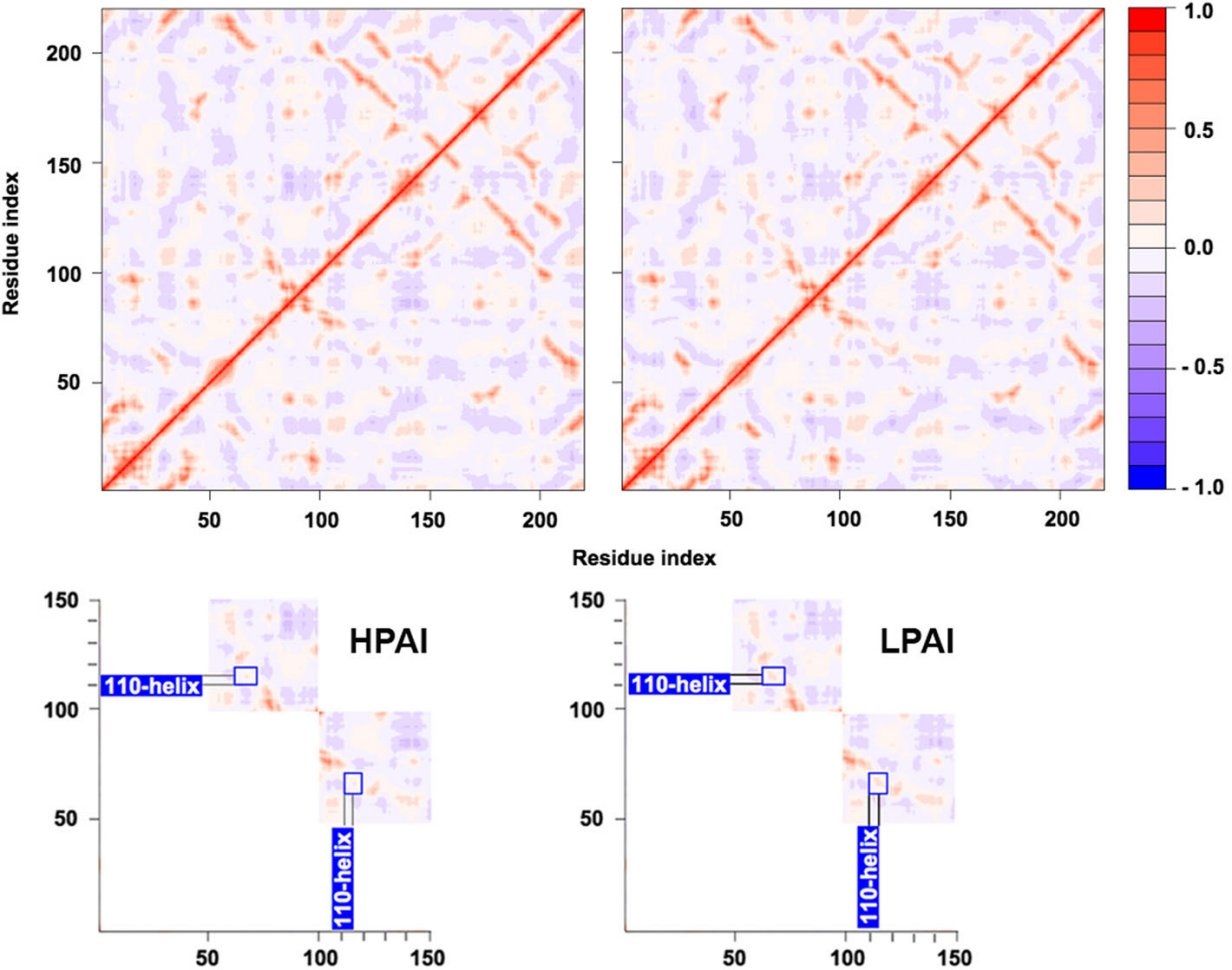
Dynamic cross-correlation matrices (DCCM) from representative HPAI and LPAI RBDs. DCCM for A0A2D9 (representative for the HPAI cluster) and A4K2G6 (representative for the LPAI cluster) are presented, where amino acid residue numbers for the proteins are reported at the x-axis and DCCM value range is colour coded at the right side, from negative correlation motions (blue) to positive correlation ones (red). In the zoom-in of 100–150 sub-area of the matrices, varying motions are boxed and related to highlighted 110-helix regions

Dynamic cross-correlation matrices (DCCMs) representations may help finding meaningful differences within a picture of overall strong conservation, as they provide an inspection of correlated and anticorrelated motions. Initially, we assessed that within each cluster, either all 10 HPAI or LPAI matrices show no meaningful difference, as suggested by the BC heatmap for RBDs. Therefore, DCCM from one representative HPAI RBD (A0A2D9) and one for LPAI (A4K2G6) were carefully compared. At a first sight, the motion patterns in HPAI and LPAI RBD DCCMs seem to be equal; however, after careful inspection, it is possible to identify slight differences concerning the extent of correlated and anticorrelated motions. For instance, the RBD subregion 24–34 exhibits anticorrelated motions with subregion 82–100, but the extension of these motions is different in HPAI and LPAI DCCMs. Such a little difference, together with others (i.e. the extension of anticorrelated motions between subregions 105–135 and 200–223) might explain the way of clustering in the BC heatmap. Subregion including positions 200–223 of the RBD is important because residues 222 and 223 are located in the 220 loop (a well known antigenic determinant) and position 222 is involved in receptor affinity [52]. However, the most evident difference concerns the 110-helix (another pivotal determinant) in the VED region, which is highlighted in Fig. 2, in the zoom-in of the two DCCMs. This is noteworthy, as the 110-helix region plays an important role in the regulation of the HA acid stability and changes in this region are involved in pathogenicity shift in H5N1 [28].

### NMA of HA RBDs from HPAI and LPAI strains: fluctuation profiles

**Fig. 3:**
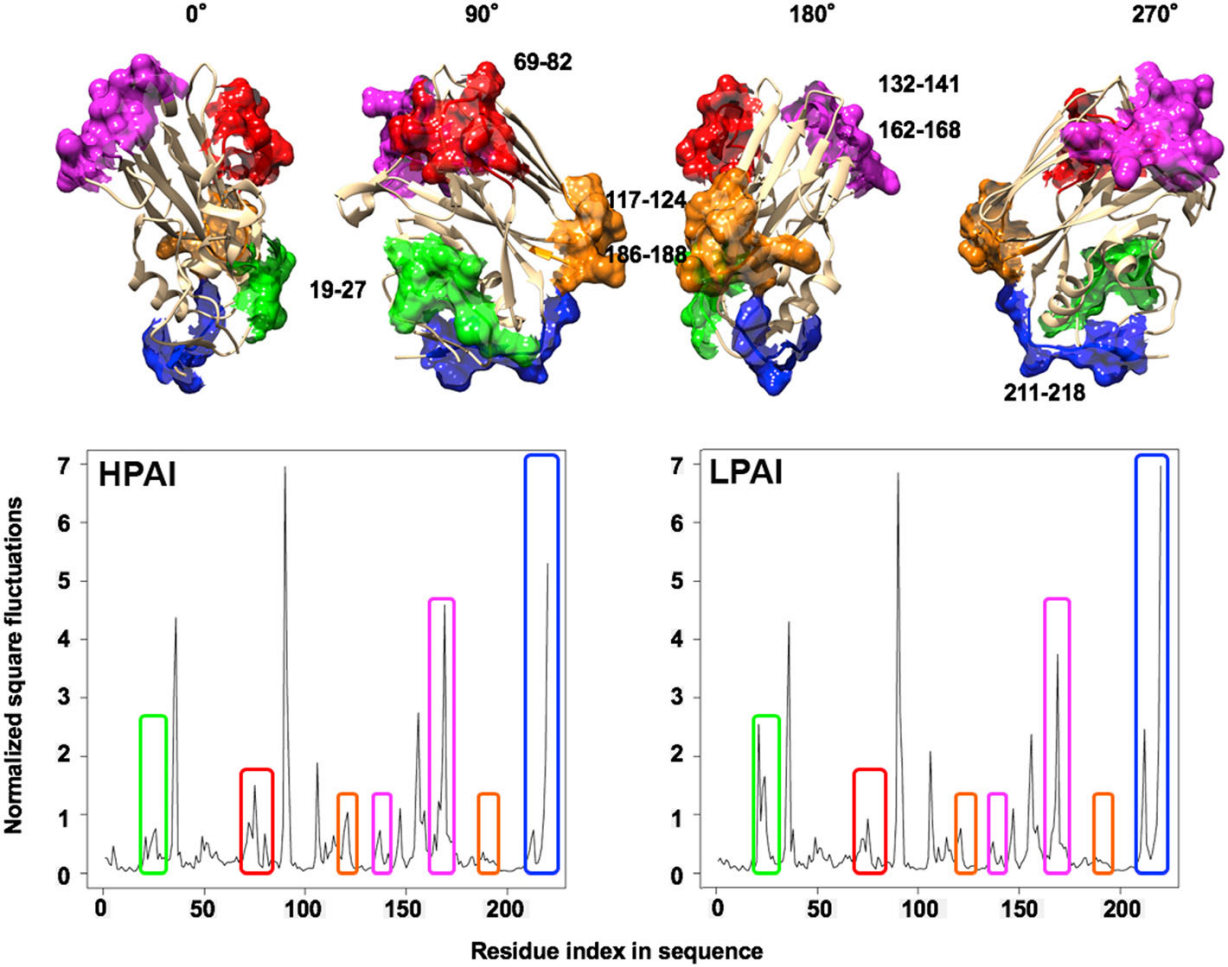
Fluctuation analysis and relationship with antigenic RBD subregions. The normalized square fluctuations plots for the same two representative RBDs shown in Fig. 2 are reported in the lower part of this figure. The upper images represent the four, 90° rotation views of the RBD, in which the antigenic regions are shown in surface format and filled with the same colors used for boxes that highlight meaningful differences in the plots

**Fig. 4:**
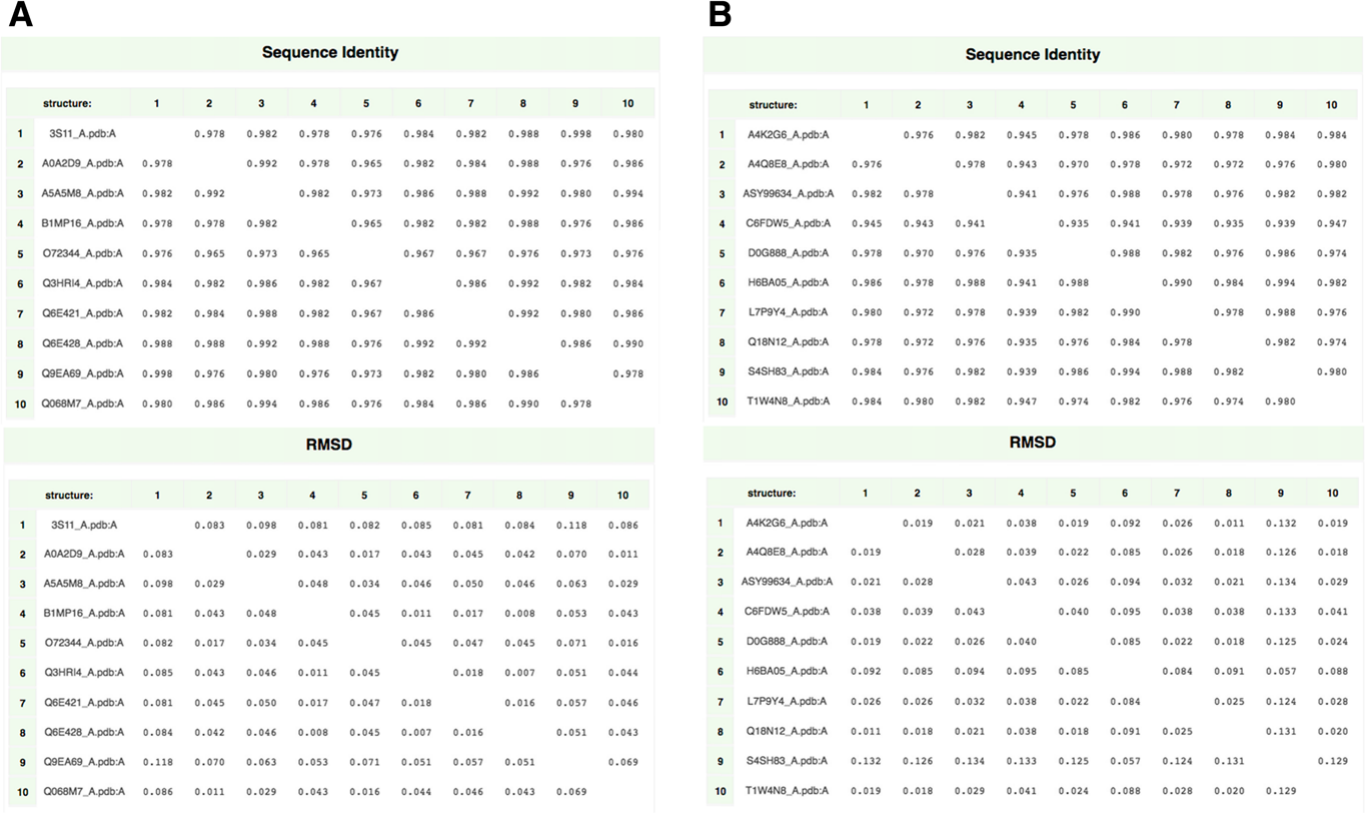
Multiple Sequence Alignment of RBD regions showing different flexibility. Sequences are identified by UniProtKb AC followed by H for HPAI and L for LPAI. Numbers for the first and last residue of each sequence block/RBD region are reported; blocks are highlighted using the same colours as boxes defining regions in Fig. 3. Changes are highlighted by bold aminoacid one-letter codes

DCCM analysis of the A0A2D9 and A4K2G6 RBDs, representative for HPAI and LPAI, respectively, was strengthened by normalized square fluctuation analysis to go deeper into subregion differences. Fluctuation profiles for the C_α_ atoms of these two HPAI and LPAI RBDs is shown in Fig. 3, and it clearly provides evidence on the difference in the amount of flexibility across the RBD sequence. It has to be stressed that antigenic subregions, such as 130-loop (134–138) and 190-helix (188–190) and residues involved in receptor specificity (136, 216) are involved in such flexibility variations [53]. Peaks variations are highlighted in different colours corresponding to the surface area plotted on the models on the top, rotated in four different 90°-step views. Repetition of the DCCM analysis with other RBDs representative for HPAI and LPAI confirmed these findings (not shown). Multiple sequence alignment (MSA) of the RDB used for the NMA analyses was performed to check whether specific aminoacid residues in regions responsible for the difference in flexibility would be conserved or not within the whole dataset or differentially conserved between HPAI and LPAI subsets. In Fig. 4, MSA blocks corresponding to regions boxed in Fig. 3 are highlighted using the same colours. Indeed, considering the overall high % identity among HPAI and LPAI RBD sequences, it was not surprising to find 100% conservation and no HPAI-LPAI difference for some of such regions, like e.g. 117–124 + 186–188, 132–141 and 211–218. Variation at the RBD positions 78 (within region 69–82) and 164 (within region 162–168) is not related as well to pathogenic type, as HPAI strain, in addition to showing E78 and K164, share D78 and E164 with LPAI strains. Seemingly, the only two aminoacid positions showing HPAI or LPAI specific residues are found in regions 19–27 (I23 for HPAI and L23 for LPAI) and 69–82 (K71 for HPAI and R71 for LPAI). This further supports the validity of the structure based investigation, as NMA could identify also regions in which HPAI and LPAI show different flexibility in spite of sequence variation inside. Once again, this is not surprising, as most features of a protein region (including flexibility) are not determined only by the corresponding sequence, while being influenced also by the local structural context, i.e. by variation in the features (steric hindrance, charge etc.) of surrounding (at three-dimensional level) elements. Highlighting relevant changes from the overall variation noise by MSA alone is often very difficult and this analyis provides further evidence on how NMA and other structure-based analyses may be of great help.

### Surface electrostatics of HA RBDs from HPAI and LPAI strains

**Fig. 5:**
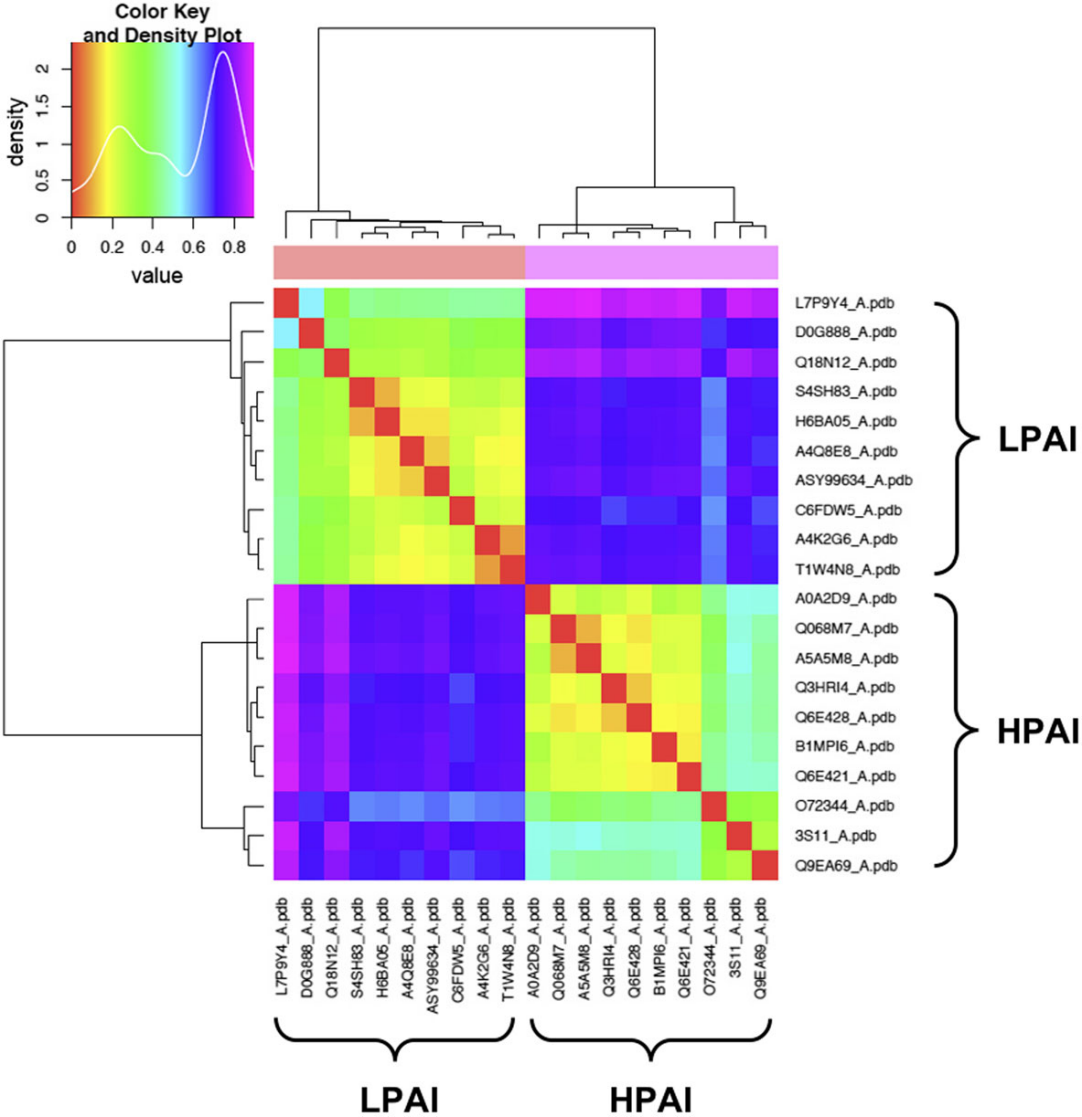
Heat map for the 10 HPAI + 10 LPAI viruses. Red, warm and cold colors correspond to identity, low and high Electrostatic Distances (ED), respectively, as reported in the ‘Density Plot’

In order to check whether sorting of HPAI and LPAI into two different clusters by NMA would be confirmed by another approach, we also investigated the 20 representative HA proteins by surface electrostatics analysis. The spatial distribution of the electrostatic potential was calculated at I = 150 mM (physiological value), assuming + 1/− 1 charges for the counter-ions. Prior to electrostatic potential calculations, partial charges and van der Waals radii were assigned with PDB2PQR [36, 37]; then, linear Poisson-Boltzmann (PB) equation calculations were carried out by using Adaptive PB Solver (APBS) [35] through Opal web service (see methods). The spatial distribution of the electrostatic potential was determined for each HA subregion. Data obtained from monomers and trimers are shown in the supplementary material. In particular, we focused on the role of charge distribution as visualized by isopotential contours within the tertiary structure, and on classifying conservation and divergence among HA subregions of HPAI and LPAI viruses. In order to evaluate the electrostatic distance (ED) also in a quantitative way, clustering of the spatial distributions of the electrostatic potentials was obtained by WebPIPSA (Protein Interaction Property Similarity Analysis) [42], using the Hodgkin and Carbo similarity index (SI) [54] (see methods). Heat maps obtained using Hodgkin SI are shown in Fig. 5; corresponding maps for the same dataset obtained with Carbo index did not show any meaningful difference and thus they are not shown. When using WebPIPSA, the distance matrix of the electrostatic potential can also be displayed as a tree referred to as ‘epogram’ (electrostatic potential diagram). We can notice a striking agreement between data obtained from NMA and results from the electrostatic analyses: once again, HPAI and LPAI RBDs are sorted in only two different clusters, each specific to the pathogenicity type.

**Fig. 6:**
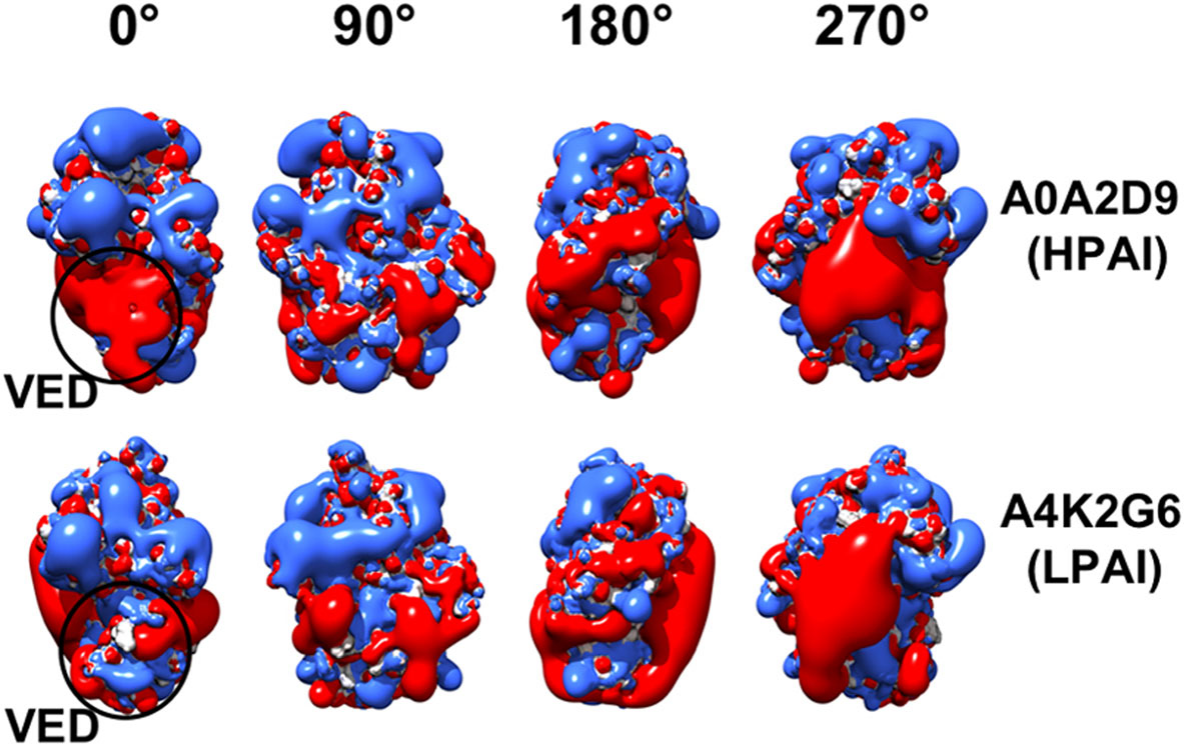
Isopotential contours for representative HPAI and LPAI RBDs. Electrostatic potentials (blue for positive and red for negative) are presented in four 90°-stepwise orientations (0° to 270°). Difference in the VED subregion is indicated

For congruence and integration with comparative NMA performed in this work, the RBDs from the aforementioned viruses representative for HPAI (A0A2D9) and LPAI (A4K2G6) were comparatively analysed also for their surface electrostatics features. Figure 6, showing four 90°- step views for the isopotential contours of the two RBDs, highlights the agreement between NMA and ED clustering. Differences between these two RBDs are very evident in the 0° view: once again, the difference is found at the VED subregion (including the 110-helix), where the isopotential contour is more negative in the HPAI virus than in the LPAI representative. Moreover, a positive potential redistribution at the 130-loop antigenic site can be observed. Differences between the aforementioned two viruses were found to confirm representativeness of the overall difference among HPAI and LPAI group by iterating this comparison with other members of the dataset (not shown). Intriguingly, the total net charge is always positive for the RBDs from HPAI viruses, ranging from 0.0000 *e* to 1.0000 *e*, whereas the charge for LPAI viruses ranges from 0.0000 *e* to − 3.0000 *e*. This cannot be explained by the well-known cluster of positively charged residues at the HA cleavage site of HPAI viruses, as this site is not part of the RBD.

## Conclusions

Influenza A viruses are under continuous and worldwide coordinated surveillance because of their huge impact on both the economy and human and animal health, especially with the occurrence of pandemic outbreaks. Therefore, several projects for pan-vaccines or universal vaccines are ongoing [22, 55]. Typisation of viral strains and studies for the unveiling, and somehow monitoring, their variation with predictive outcome are very important. For several decades, the study of AI viruses variation has been mainly based on sequencing (in the most recent decade, strongly enhanced by next-generation techniques) followed by phylogenetic analyses, and on serological characterisation. Some fingerprints could be found, as presented and discussed in the introduction section after a number of haemagglutinin protein structures were solved and mutants analysed. In recent years, we provided an alternative approach to the study of viral variation, based on the deep dissection of the surface features of the main spike protein haemagglutinin [23–25]. This led us to demonstrate that variation of surface electrostatics features is a fingerprint for both viral clade evolution and spreading in H5N1 viruses, and then this was confirmed to be a hallmark for AI viruses by comparative analysis of H9N2 strains. After finding that variation in the surface features of the main spike protein of AI viruses is tightly related to their evolution and spreading, as well as to antigenic drift, it was not surprising to find that electrostatics is also involved (even if it is not the main determinant) in host jump. Indeed, the biology of an organism depends on its interactions with others and the micro- and macro-environment. This prompted us to further investigate by following a similar approach the last, but not least important ‘shifting’ phenomenon in AI virus biology: the LPAI to HPAI pathogenicity shift. Indeed, in addition to electrostatics comparison, we also used NMA to gain insights into almost ‘hidden’ differences that could not be captured by simple sequence and stucture comparison or other analytic systems. Evidence in this work clearly demonstrates that specific fingerprints for HPAI and LPAI viruses can be found and that the two, independent approaches followed in this work confirm such specific clustering. More importantly, this work suggests that the VED subregion of the RBD, and in particular the 110-helix subregion (already candidate by other studies for playing a special role in pathogenicity shift, as discussed in the introduction section), might play a pivotal role in the dangerous outbreak of HPAI strains [56] and thus, in addition to the poly-basic cluster at the cleavage site, it could become an important fingerprint for pathogenic virus classification and surveillance, as well as for vaccine design.

## Supplementary information

**Additional file 1: Table S1.** Pathogenicity (HPAI or LPAI), name (based on year and location of identification), UniProtKb accession number (Ac) and relevant reference are reported for each strain.

**Additional file 2: Figure S1.** Identity and RMSD values across HPAI and LPAI trimers. Values are comparable to those obtained from RBDs

**Additional file 3: Figure S2.** Bhattacharyya coefficient (BC) heatmap for HPAI and LPAI Monomers. The Uniprot AC (and only one PDB AC) of theproteins are reported at both the x- and y-axis, while BC value range in figure is colour coded from the lowest (blue) to the highest (red). **Figure S3.** Bhattacharyya coefficient (BC) heatmap for HPAI and LPAI Trimers. The Uniprot AC (and only one PDB AC) of the proteins are reported at both the x- and y-axis, while BC value range in figure is colour coded from the lowest (blue) to the highest (red). **Figure S4.** Bhattacharyya coefficient (BC) heatmap. Repetition of NMA after modelling all twenty HPAI and LPAI target sequences on the LPAI structural template.

## Data Availability

Not applicable.

## Abbreviations

AC: Accession code
AI: Avian influenza
BC: Bhattacharyya coefficient
APBS: Adaptive PB Solver
CDC: Center for Disease Control
DCCM: Dynamic cross-correlation matrix
ED: Electrostatic distance
ENM: Elastic network model
Epogram: Electrostatic potential diagram
HA: Haemagglutinin
HPAI: High pathogenic AI
I: Ionic strength
LPAI: Low pathogenic AI
N: Neuraminidase
NMA: Normal Modes Analysis
PB: Poisson-Boltzmann
PDB: Protein Data Bank
PIPSA: Protein Interaction Property Similarity Analysis
RBD: Receptor-binding domain
RMSD: Root Mean Square Deviation
RMSIP: Root Mean Square Inner Product
SI: Similarity index
VED: Vestigial esterase domain
WHO: World Health Organization
