# Supplementary material for: Normal modes analysis and surface electrostatics of haemagglutinin proteins as fingerprints for high pathogenic type A influenza viruses
Source: BMC Bioinformatics. 2020 Aug 21;21(Suppl 10):354. doi: 10.1186/s12859-020-03563-w (PMC7445075; doi:10.1186/s12859-020-03563-w)
Supplement: Supplementary file 1 — Additional file 1: Table S1. Pathogenicity (HPAI or LPAI), name (based on year and location of identification), UniProtKb accession number (Ac) and relevant reference are reported for each strain. [file 12859_2020_3563_MOESM1_ESM.docx]

**Supplementary table S1.** Pathogenicity (HPAI or LPAI), name (based on year and location of identification), UniProtKb accession number (Ac) and relevant reference are reported for each strain.

| **PAI** | **viral strain** | **UniProtKB Ac** | **Reference** |
| --- | --- | --- | --- |
| HPAI | A/environment/Hong Kong/437-4/99 | Q9EA69 | S1 |
| HPAI | A/goose/Shantou/5456/2001 | B1MPI6 | S2 |
| HPAI | A/duck/Guangdong/01/2001 | Q6E428 | S3 |
| HPAI | A/Goose/Huadong/1/2000 | Q3HRI4 | S4 |
| HPAI | A/duck/Shanghai/13/2001 | Q6E421 | S5 |
| HPAI | A/duck/Viet Nam/Ncvd1/2003 | A5A5M8 | S6 |
| HPAI | A/Chicken/Hong Kong/258/97 | O72344 | S7 |
| HPAI | A/duck/Shanghai/xj/2002 | A0A2D9 | S8 |
| HPAI | A/chicken/Jiangsu/cz1/2002 | Q068M7 | S9 |
| HPAI | A/Goose/Guangdong/1/1996 | Q9Q0U6  (PDB 3S11) | S10 |
| LPAI | A/mallard/Italy/3401/2005 | A4K2G6 | S11 |
| LPAI | A/duck/France/05066b/2005 | A4Q8E8 | S12 |
| LPAI | A/mallard duck/Netherlands/41/2015 | A0A2U7NY21  (ASY99634) | S13 |
| LPAI | A/turkey/Ontario/84/1983 | C6FDW5 | S14 |
| LPAI | A/mallard/Hokkaido/24/2009 | D0G888 | S15 |
| LPAI | A/wild duck/Korea/CSM4-12/2009 | H6BA05 | S16 |
| LPAI | A/wild duck/Korea/SNU50-5/2009 | L7P9Y4 | S17 |
| LPAI | A/R(duck/Mongolia/54/01-duck/Mongolia/47/01 | Q18N12 | S18 |
| LPAI | A/wild bird/Korea/A344-2/2009 | S4SH83 | S19 |
| LPAI | A/European teal/Novosibirsk/203/2011 | T1W4N8 | S20 |
|  |  |  |  |
|  |  |  |  |

**REFERENCES**

S1. Cauthen AN, Swayne DE, Schultz-Cherry S, Perdue ML, Suarez DL: Continued circulation in China of highly pathogenic avian influenza viruses encoding the hemagglutinin gene associated with the 1997 H5N1 outbreak in poultry and humans. J Virol 2000, 74(14):6592-6599.

S2. Wang J, Vijaykrishna D, Duan L, Bahl J, Zhang JX, Webster RG, Peiris JS, Chen H, Smith GJ, Guan Y: Identification of the progenitors of Indonesian and Vietnamese avian influenza A (H5N1) viruses from southern China. J Virol 2008, 82(7):3405-3414.

S3. Steensels M, Van Borm S, Boschmans M, van den Berg T: Lethality and molecular characterization of an HPAI H5N1 virus isolated from eagles smuggled from Thailand into Europe. Avian Dis 2007, 51(1 Suppl):401-407.

S4. Zhu X, Guo YH, Jiang T, Wang YD, Chan KH, Li XF, Yu W, McBride R, Paulson JC, Yuen KY, Qin CF, Che XY, Wilson IA: A unique and conserved neutralization epitope in H5N1 influenza viruses identified by an antibody against the A/Goose/Guangdong/1/96 hemagglutinin. J Virol 2013, 87(23):12619-12635.

S5. Guo XL, Zhu YS, Li YX, Shi P, Zhou HK, Yao JS, Huang ZD, Wei DQ: Genetic insight of the H5N1 hemagglutinin cleavage site. Chin Sci Bull 2007, 52:2374.

S6. Jadhao SJ, Nguyen DC, Uyeki TM, Shaw M, Maines T, Rowe T, Smith C, Huynh LP, Nghiem HK, Nguyen DH, Nguyen HK, Nguyen HH, Hoang LT, Nguyen T, Phuong LS, Klimov A, Tumpey TM, Cox NJ, Donis RO, Matsuoka Y, Katz JM: Genetic analysis of avian influenza A viruses isolated from domestic waterfowl in live-bird markets of Hanoi, Vietnam, preceding fatal H5N1 human infections in 2004. Arch Virol 2009, 154(8):1249-1261.

S7. Claas EC, Osterhaus AD, van Beek R, De Jong JC, Rimmelzwaan GF, Senne DA, Krauss S, Shortridge KF, Webster RG: Human influenza A H5N1 virus related to a highly pathogenic avian influenza virus. Lancet 1998, 351(9101):472-477.

S8. Bragstad K, Jørgensen PH, Handberg K, Hammer AS, Kabell S, Fomsgaard A: First introduction of highly pathogenic H5N1 avian influenza A viruses in wild and domestic birds in Denmark, Northern Europe. Virol J 2007, 4:43.

S9. Ozawa M, Matsuu A, Tokorozaki K, Horie M, Masatani T, Nakagawa H, Okuya K, Kawabata T, Toda S: Genetic diversity of highly pathogenic H5N8 avian influenza viruses at a single overwintering site of migratory birds in Japan, 2014/15. Euro Surveill 2015, 20(20) pii:21132.

S10. Velkov T, Ong C, Baker MA, Kim H, Li J, Nation RL, Huang JX, Cooper MA, Rockman S: The antigenic architecture of the hemagglutinin of influenza H5N1 viruses. Mol Immunol 2013, 56:705-719.

S11. Fusaro A, Monne I, Cattoli G, De Nardi R, Salviato A, Moreno Martin A, Capua I, Terregino C: Gene segment reassortment between Eurasian and American clades of avian influenza virus in Italy. Arch Virol 2010, 155(1):77-81.

S12. Briand FX, Le Gall-Reculé G, Guillou-Cloarec C, Ogor K, Jestin V: Phylogeny and genotyping of recent avian low-pathogenic H5 subtype influenza viruses from French ducks. J Gen Virol 2010, 91(Pt 4):960-970.

S13. Poen MJ, Verhagen JH, Vuong O, Scheuer RD, Pas SD, Fouchier RAM: Centers of Excellence for Influenza Research and Surveillance (CEIRS) Direct Submission Submitted (22-AUG-2017) Center for Research on Influenza Pathogenesis (CRIP), New York, NY 10029-6574, USA.

S14. Pei Y, Swinton J, Ojkic D, Sharif S: Genetic characterization of two low pathogenic avian influenza virus H5N1 isolates from Ontario, Canada. Virus Genes 2009, 38(1):149-154.

S15. Yamamoto N, Sakoda Y, Motoshima M, Yoshino F, Soda K, Okamatsu M, Kida H:

Characterization of a non-pathogenic H5N1 influenza virus isolated from a migratory duck flying from Siberia in Hokkaido, Japan, in October 2009. Virol J 2011, 8:65.

S16. Kim BS, Kang HM, Choi JG, Kim MC, Kim HR, Paek MR, Kwon JH, Lee YJ: Characterization of the low-pathogenic H5N1 avian influenza virus in South Korea. Poult Sci 2011, 90(7):1449-1461.

S17. Kim IH, Kwon HJ, Choi JG, Kang HM, Lee YJ, Kim JH: Characterization of mutations associated with the adaptation of a low-pathogenic H5N1 avian influenza virus to chicken embryos. Vet Microbiol 2013, 162(2-4):471-478.

S18. Isoda N, Sakoda Y, Kishida N, Soda K, Sakabe S, Sakamoto R, Imamura T, Sakaguchi M, Sasaki T, Kokumai N, Ohgitani T, Saijo K, Sawata A, Hagiwara J, Lin Z, Kida H: Potency of an inactivated avian influenza vaccine prepared from a non-pathogenic H5N1 reassortant virus generated between isolates from migratory ducks in Asia. Arch Virol 2008, 153(9):1685-1692.

S19. Kim HR, Oem JK, Bae YC, Kang MS, Lee HS, Kwon YK: Application of real-time reverse transcription polymerase chain reaction to the detection the matrix, H5 and H7 genes of avian influenza viruses in field samples from South Korea. Virol J 2013, 10:85.

S20. Briand FX, Schmitz A, Ogor K, Le Prioux A, Guillou-Cloarec C, Guillemoto C, Allée C, Le Bras MO, Hirchaud E, Quenault H, Touzain F, Cherbonnel-Pansart M, Lemaitre E, Courtillon C, Gares H, Daniel P, Fediaevsky A, Massin P, Blanchard Y, Eterradossi N, van der Werf S, Jestin V, Niqueux E: Emerging highly pathogenic H5 avian influenza viruses in France during winter 2015/16: phylogenetic analyses and markers for zoonotic potential. Euro Surveill 2017, 22(9) pii:30473.
