# Supplementary material for: Normal modes analysis and surface electrostatics of haemagglutinin proteins as fingerprints for high pathogenic type A influenza viruses
Source: BMC Bioinformatics. 2020 Aug 21;21(Suppl 10):354. doi: 10.1186/s12859-020-03563-w (PMC7445075; doi:10.1186/s12859-020-03563-w)
Supplement: Supplementary file 2 — Additional file 2: Figure S1. Identity and RMSD values across HPAI and LPAI trimers. Values are comparable to those obtained from RBDs [file 12859_2020_3563_MOESM2_ESM.docx]

**Figure S1: Identity and RMSD values across HPAI and LPAI trimers.** Values are comparable to those obtained from RBDs
