# Supplementary material for: Normal modes analysis and surface electrostatics of haemagglutinin proteins as fingerprints for high pathogenic type A influenza viruses
Source: BMC Bioinformatics. 2020 Aug 21;21(Suppl 10):354. doi: 10.1186/s12859-020-03563-w (PMC7445075; doi:10.1186/s12859-020-03563-w)
Supplement: Supplementary file 3 — Additional file 3: Figure S2. Bhattacharyya coefficient (BC) heatmap for HPAI and LPAI Monomers. The Uniprot AC (and only one PDB AC) of theproteins are reported at both the x- and y-axis, while BC value range in figure is colour coded from the lowest (blue) to the highest (red). Figure S3. Bhattacharyya coefficient (BC) heatmap for HPAI and LPAI Trimers. The Uniprot AC (and only one PDB AC) of the proteins are reported at both the x- and y-axis, while BC value range in figure is colour coded from the lowest (blue) to the highest (red). Figure S4. Bhattacharyya coefficient (BC) heatmap. Repetition of NMA after modelling all twenty HPAI and LPAI target sequences on the LPAI structural template. [file 12859_2020_3563_MOESM3_ESM.docx]

**
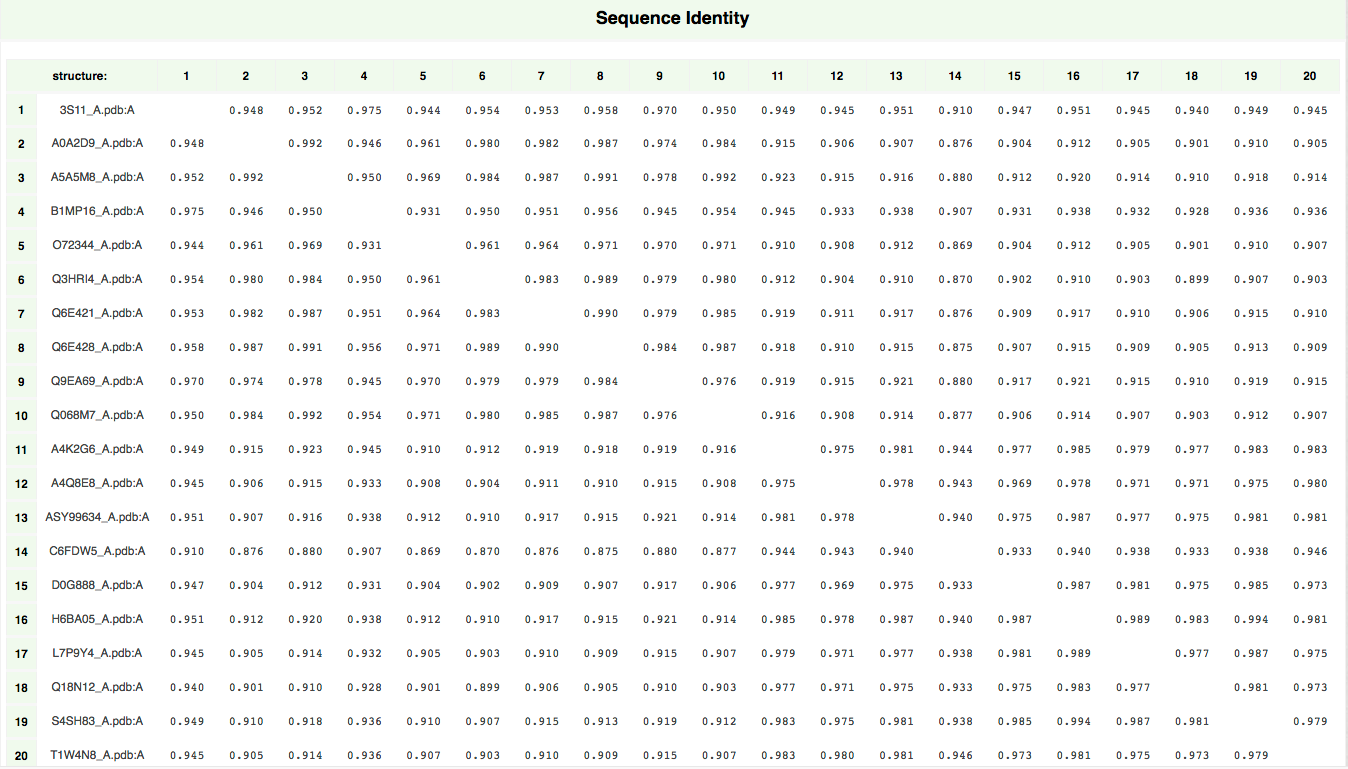

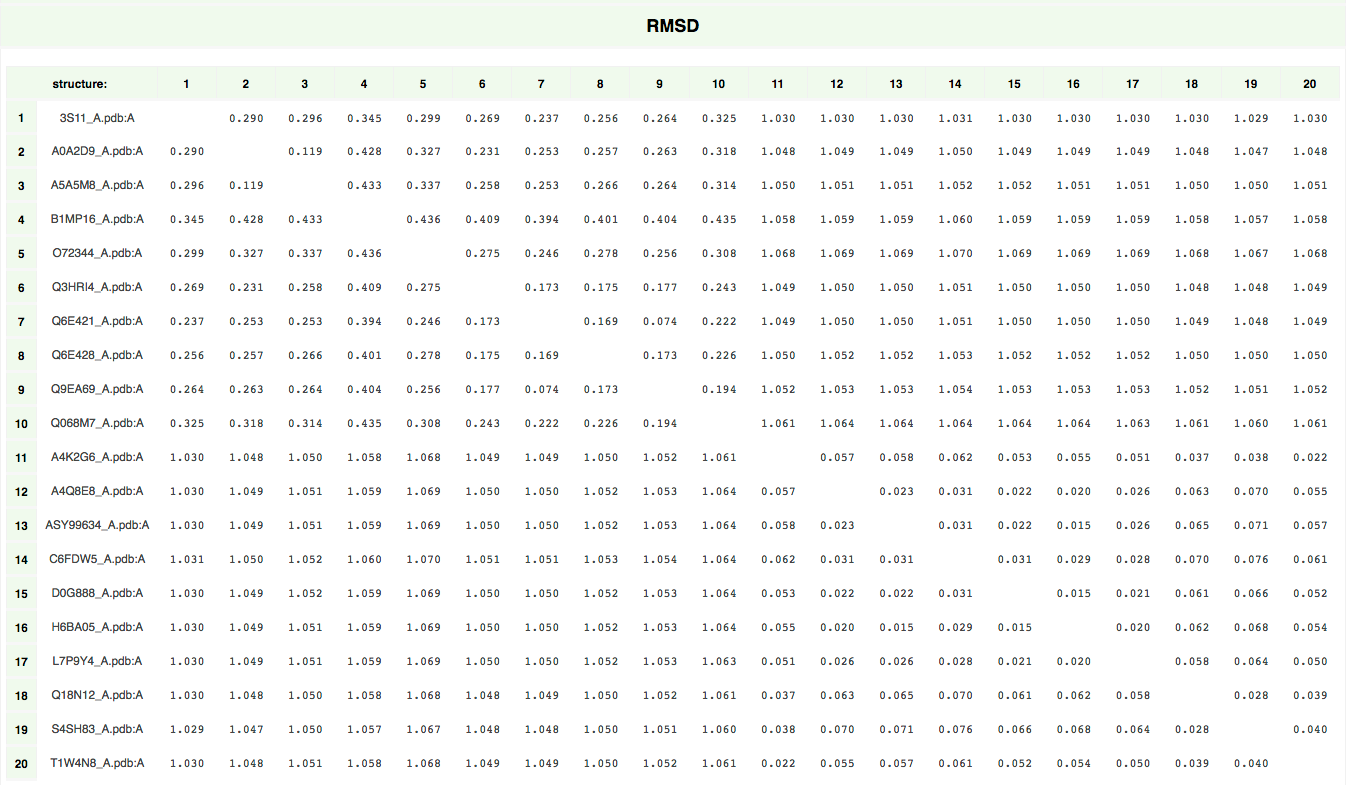
**

**Figure S2: Identity and RMSD values across HPAI and LPAI trimers.** Values are comparable to those obtained from RBDs

Figure S3: Bhattacharyya coefficient (BC) heatmap for HPAI and LPAI Monomers. The Uniprot AC (and only one PDB AC) of the proteins are reported at both the x- and y-axis, while BC value range in figure is colour coded from the lowest (blue) to the highest (red).

Figure S4: Bhattacharyya coefficient (BC) heatmap for HPAI and LPAI Trimers. The Uniprot AC (and only one PDB AC) of the proteins are reported at both the x- and y-axis, while BC value range in figure is colour coded from the lowest (blue) to the highest (red).
